# A biphasic pattern of gene expression during mouse retina development

**DOI:** 10.1186/1471-213X-6-48

**Published:** 2006-10-17

**Authors:** Samuel Shao-Min Zhang, Xuming Xu, Mu-Gen Liu, Hongyu Zhao, Marcelo Bento Soares, Colin J Barnstable, Xin-Yuan Fu

**Affiliations:** 1 Departments of Ophthalmology and Visual Science, Yale School of Medicine, New Haven, Connecticut, USA; 2 Epidemiology and Public Health and Genetics, Yale School of Medicine, New Haven, Connecticut, USA; 3 Children's Memorial Research Center, Northwestern University's Feinberg School of Medicine, Illinois, USA; 4 Department of Microbiology & Immunology, Indiana University School of Medicine, Indiana, USA; 5 Department of Neural and Behavioral Sciences, Pennsylvania State University College of Medicine, Hershey, Pennsylvania, USA

## Abstract

**Background:**

Between embryonic day 12 and postnatal day 21, six major neuronal and one glia cell type are generated from multipotential progenitors in a characteristic sequence during mouse retina development. We investigated expression patterns of retina transcripts during the major embryonic and postnatal developmental stages to provide a systematic view of normal mouse retina development,

**Results:**

A tissue-specific cDNA microarray was generated using a set of sequence non-redundant EST clones collected from mouse retina. Eleven stages of mouse retina, from embryonic day 12.5 (El2.5) to postnatal day 21 (PN21), were collected for RNA isolation. Non-amplified RNAs were labeled for microarray experiments and three sets of data were analyzed for significance, hierarchical relationships, and functional clustering. Six individual gene expression clusters were identified based on expression patterns of transcripts through retina development. Two developmental phases were clearly divided with postnatal day 5 (PN5) as a separate cluster. Among 4,180 transcripts that changed significantly during development, approximately 2/3 of the genes were expressed at high levels up until PN5 and then declined whereas the other 1/3 of the genes increased expression from PN5 and remained at the higher levels until at least PN21. Less than 1% of the genes observed showed a peak of expression between the two phases. Among the later increased population, only about 40% genes are correlated with rod photoreceptors, indicating that multiple cell types contributed to gene expression in this phase. Within the same functional classes, however, different gene populations were expressed in distinct developmental phases. A correlation coefficient analysis of gene expression during retina development between previous SAGE studies and this study was also carried out.

**Conclusion:**

This study provides a complementary genome-wide view of common gene dynamics and a broad molecular classification of mouse retina development. Different genes in the same functional clusters are expressed in the different developmental stages, suggesting that cells might change gene expression profiles from differentiation to maturation stages. We propose that large-scale changes in gene regulation during development are necessary for the final maturation and function of the retina.

## Background

A dynamic process of retina differentiation occurs from embryonic day 12 to postnatal day 21. Six major neuronal and one glia cell type are generated from multipotential progenitors in a characteristic sequence during development [1-4]. Revealing the intrinsic program of gene regulation that accompanies mammalian retinal development is a key step for eventual cure of many human retina diseases and blindness.

Microarray analysis has been used extensively in studies of tumor biology [5-7] as well as transcription and genome organization in yeast [8-13]. In the eye there have been a number of microarray studies but few have studied changes in gene expression over development [14-17]. Many of the previous studies focused on a specific cell type, for example using isolated target single cell [18] or cell populations [19], or retinas from cell type specific mutants [20-23]. Gene expression profiles obtained without artifacts introduced by isolation procedures or gene mutations provide a valuable overview of retina development under natural developmental conditions.

There are several reasons for the slow adoption of microarray technology to studies of development, especially in mammals, [24]. First, temporally and spatially restricted tissue or cell patterns cause difficulties in dissecting target tissues or cells to collect enough RNA. Second, a broader spectrum of experimental points during developmental processes leads to increased costs, especially when using common comprehensive arrays. Third, even using currently available comprehensive arrays, tissue-specific genes are still under-represented, including alternatively spliced variants or genes that are only briefly expressed in a tissue-specific pattern. In our recent report [21], about 40% of mouse retina transcripts are not contained within the comprehensive set of RIKEN 60,770 mouse full-length cDNAs [25], indicating that generation of a tissue-specific microarray might be helpful to make complete gene expression profiles for specific tissues or functional units.

In this study we have used whole retinal RNA, without amplification, to obtain an overall view of gene expression during retina development that is not restricted to individual genes or retinal cell types. We found that two major groups of gene expression clusters are separated by a gene expression transition stage from PN3 to PN5. Distinct gene populations were expressed before and after this critical stage. The different genes that belong to the same functional clusters are expressed in the two groups, respectively. We suggest that a change in expression of cell cycle and chromatin modification genes results in a large change in gene expression profile and that this initiates the final maturation of the postmitotic retina.

## Results

### High performance of mouse retina tissue-specific cDNA microarrays

**Figure 1:**
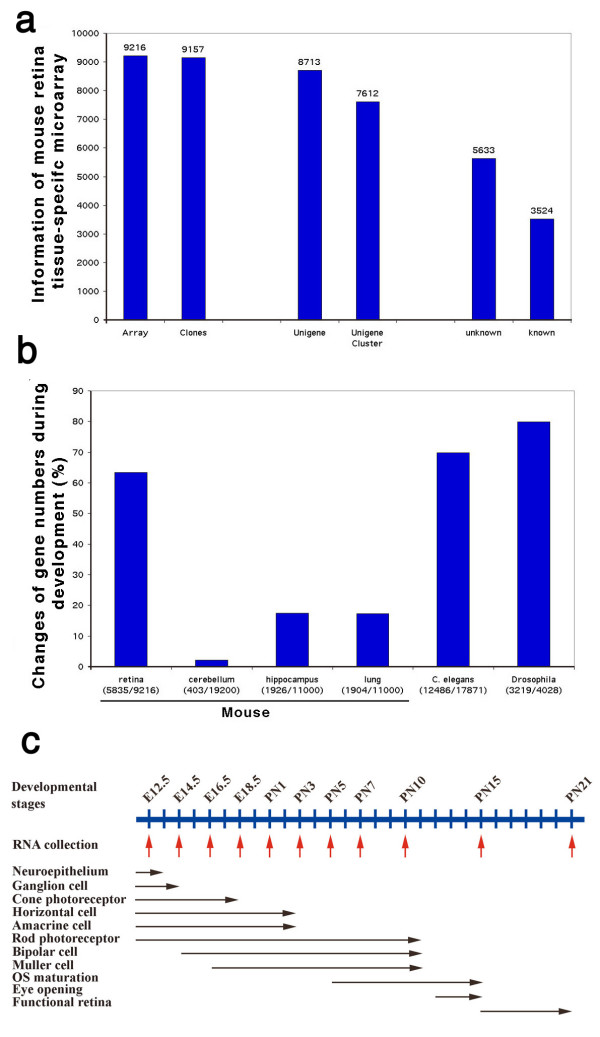
Retina tissue-specific cDNA microarray and gene expression clusters during mouse retina development. a. Composition of retina tissue-specific cDNA microarray. b. Microarray experimental comparison among Drosophila, C. elegans, and distinct mammalian tissues by using different microarray. c. Cell differentiation profiles during mouse retina development and microarray experimental design (text in details).

We have constructed a novel retina tissue-specific cDNA microarray that contains over 9,100 clones representing more than 7,600 UniGene clusters (Fig. 1a) [21]. Tissue was collected at E12.5 (embryonic day 12.5), E14.5, E16.5, and E18.5 as well as PN1 (postnatal day 1), PN3, PN5, PN7, PN10, PN15, and PN21 (Fig. 1c) and pools of 20 to 200 retinas from each time point were used to cover the whole period of retinogenesis. Total RNA (5 μg) from each time point was used for each microarray reaction without amplification. A reference sample consisting of equal amounts of RNA from all time points was used as a normalization control for each experiment. Three microarrays were processed for each time point and data points showing differences from control that were both log_2 _> 1 and P < 0.05 in a one-way ANOVA test were collected for further analysis. The list of average expression values of each gene at all time points is provided as additional file 1.

In the present study, about 70% (6,689) of genes showed at least a 2-fold difference in expression during development (Fig. 1b). Genes were designated as changed significantly if the average changes from the three arrays were greater than 2-fold with p < 0.05 by one-way ANOVA analysis. The ratios reflected the changes of the gene expression compared with its normalized averages. The actual alteration of the gene expression was much bigger between each time point. For example, clone BE950188 annotated as Rhodopsin had a ratio 0.01 at E12.5 and a ratio 3.15 at PN21, giving an actual difference of 315 fold between the two time points. A total of 4,180 (45%) genes met both criteria, and a full list of changed genes is shown in additional file 2. This proportion is higher than the 20% of genes that were found to be changed in previous studies of mouse development using cerebellum, hippocampus, and lung [26-28]. It is, however, similar to the 70% to 80% of genes that changed significantly during the developmental survey period in Drosophila [29] and C. elegans [30].

Some of the genes we found to change during development have been previously studied using quantitative RT-PCR (qPCR) and in situ hybridization. We have previously shown a similar ratio and expression pattern of mouse membrane palmitoylated protein 4 (Mpp4) gene by qPCR and microarray analysis [31]. The comparison of qPCR and microarray results for Rho, Rds, Glu1, Ccnd1, Ccpg1, Cdk2ap1, Ext2, Fh11, Tmpo, and Sat1 genes are also shown in additional file 3.

### Classification of development stage by hierarchical clustering

**Figure 2:**
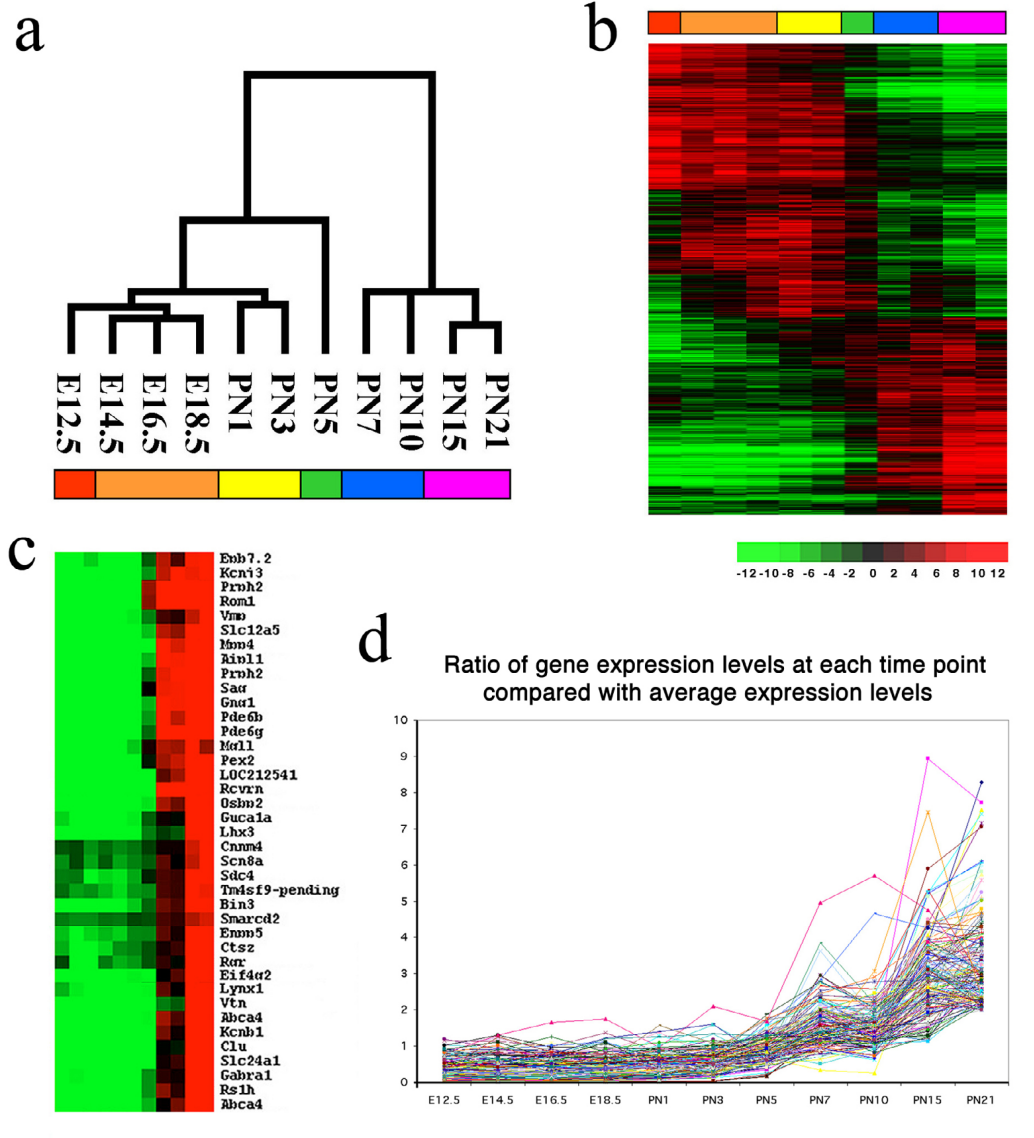
Gene expression clustering during retina development. a. Tree-chart of correlative gene expression patterns from gene cluster analysis. b. Imaging of gene classification of retina development through hierarchical clustering. c. Sample gene expression patterns from functional phase of later developmental stages. d. 130 later expressed genes during retina development

Eisen's program [9] was used to cluster gene expression at distinct retina developmental points. Figure 2a shows the tree-chart of correlative gene expression from E12.5 to PN21. Two development phases are evident: a "developmental phase" (from E12.5 to PN5) and a "functional phase" (from PN7 on) based on their correlative distance of gene expression. An obvious gap of gene expression patterns between the two phases is the transition stage PN5 in all the observations of this study. This is in agreement with a recent observation [32] that the starting point of outer plexiform layer formation might be the transition between stages of cell commitment and maturation and occurs at about PN5.

Six expression clusters were also defined through the period of development studied. In the "developmental phase", there are four clusters: E12.5 (Cluster-I, neuroepithelial stage), E14.5 to E18.5 (Cluster-II, early-born neuron differentiation stage), PN1 to PN3 (Cluster-III, later-born neuron differentiation stage), and PN5 (Cluster-IV, transition stage). Many genes are expressed in only one or two clusters. For example, high expression of Igf2, Igf2bp1, and Six6 were found in Cluster-I; Grb10, Ccnb1, and Foxn4 are highly expressed in both Cluster-I and II; Fgf15 and Gap43 are highly expressed in Cluster-II; Ddx5, Hmgb3, and Kdd3 are highly expressed in Cluster-III (additional file 4). A number of genes encoding ribosomal proteins are highly expressed in Clusters-I and II (additional file 4).

In the "functional phase", two clusters, PN7 to PN10 (Cluster-V, maturation stage) and PN15 to PN21 (Cluster-VI, light response stage) were defined. Figure 2b shows gene expression profiles ordered by onset of their first increase in transcript abundance. A group of about 170 genes in the "functional phase" (additional file 5) contained major retina specific genes like Rhodopsin, Sag, Peripherin2, Abca4, Rom1, Rs1h, Rgr, Gng1, Pde6b, and Pde6g (Fig. 2c) and present a similar pattern of gene expression (Fig. 2d). The molecular classification of retinal development through hierarchical clustering on the basis of gene expression patterns correlates well with the previous classification of retina development from the studies of morphology and biochemistry.

### Functional views of retina developmental clusters

**Figure 3:**
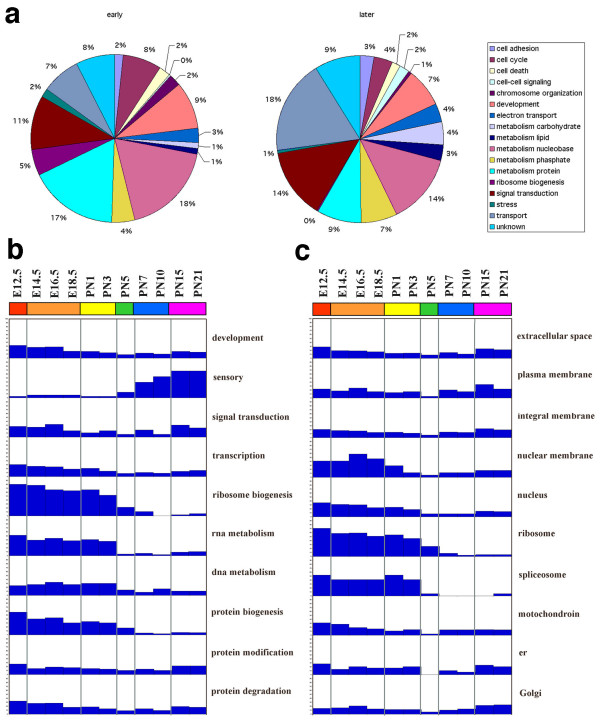
Functional clusters and classification of genes during retina development. a. GO analysis in developmental and functional phases. 17 categories are used for calculation of proportion. b. Distribution of gene expression within the sub-category biological process during retina development. c. Distribution of gene expression within the sub-category cellular components during retina development.

To test whether there were differences in gene functions between the "developmental phase" and the "functional phase", the Gene Ontology (GO) database [33] was used to assign expressed genes to functional groups. 17 sub-categories under *biological processes *were employed to analyze the gene functions in the two phases. The relative proportions of these functional groups in those two phases are shown in the pie charts of Figure 3a. The sub-categories, *protein metabolism, cell cycle, cell-cell signaling, chromosome organization *and *stress response*, constituted a 2-fold greater proportion in the "developmental phase" than in the "functional phase". *Ribosome biogenesis *showed an even greater difference, constituting 5% of the "developmental phase" but only 0.1% of the "functional phase". Conversely, *carbohydrate metabolism, lipid metabolism, phosphate metabolism*, and *transport *functional groups all represented a significantly increased proportion in the "functional phase" compared with the "developmental phase". There were no changes in *cell adhesion, cell death, development, nucleotide metabolism, signal transduction *and *functional unknown groups *in the two phases.

These changes are shown in more detail in Figures 3b and 3c using the *cellular component *category of the GO database in addition to the *biological processes *category. The percentage of genes with higher expression levels (log_2 _>= 1) in each functional group was calculated at each age. In the *ribosome biogenesis, RNA metabolism*, and *protein biogenesis *groups (Fig. 3b), more than 50% of genes showed elevated expression in the "developmental phase", but less than 10% in the "functional phase". The percentage of genes expressed at higher levels in the first three cluster periods (from E12.5 to PN3) varied very little. In the *sensory function *sub-category (Fig. 3b), the percentage of genes with higher expression profiles increased gradually in the "functional phase" and maintained their higher levels in the clusters of PN15 to PN21. In other categories, such as *development, signal transduction, transcription, nucleotide metabolism, protein modification*, and *protein degradation*, the percentages of genes expressed were maintained at the same levels during the period surveyed. Using the *Cellular component *category we found that more than 50% of genes with increased expression levels were located in the nuclear membrane, nucleus, ribosome, and spliceosome groups in the period of the "developmental phase" (Fig. 3c).

### Differential expression of genes in the same functional categories

**Figure 4:**
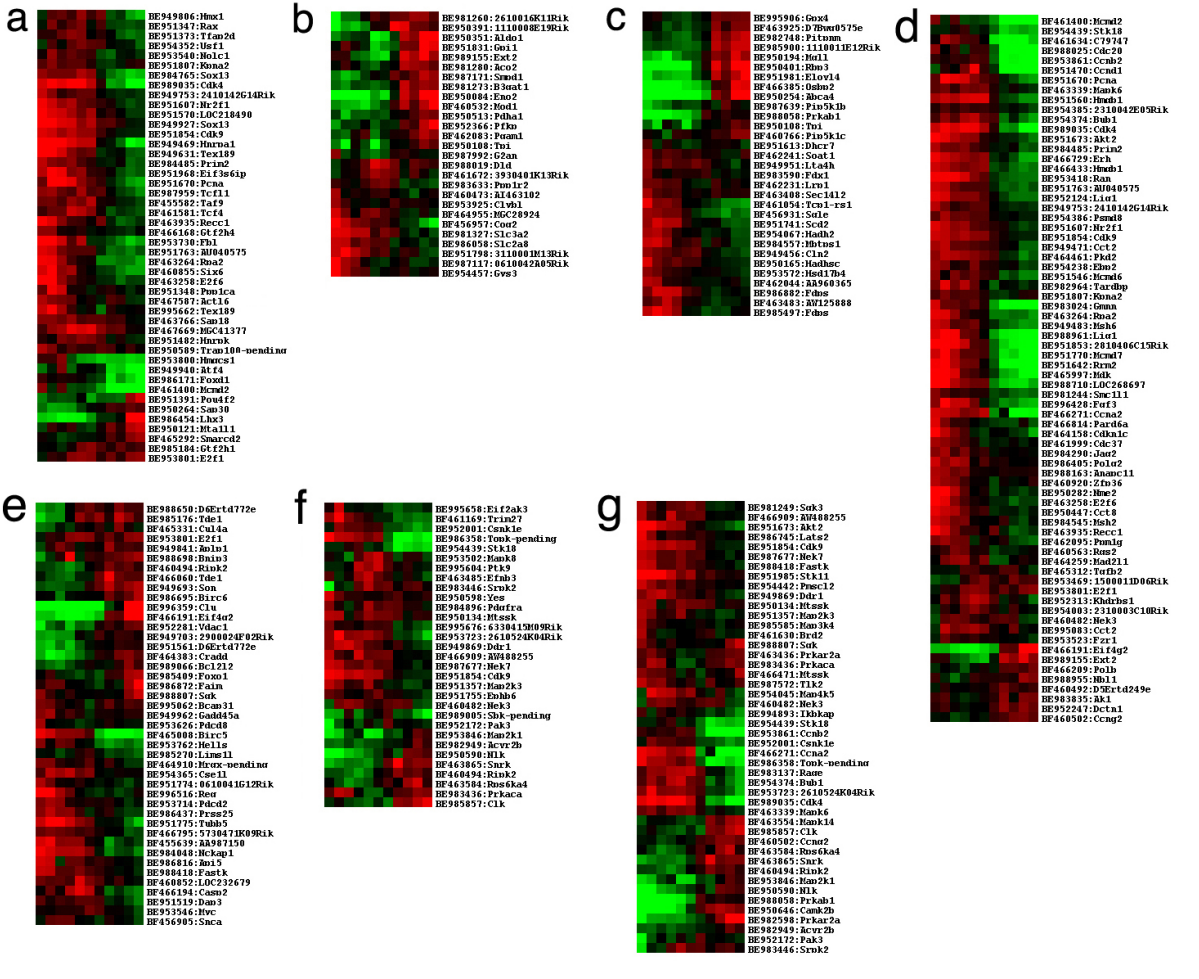
Gene differential expression in same functional clusters. a. Clustering of nuclei gene expression. b. Clustering of carbohydrate metabolism gene expression. c. Clustering of lipid metabolism gene expression. d. Clustering of cell cycle control gene expression. e. Clustering of cell death processes gene expression. f. Clustering of protein tyrosine phosphorylation gene expression. g. Clustering of protein Ser/Thr phosphorylation gene expression.

Many of the functional categories seemed to show no differences in expression throughout development. To analyze whether this broad classification might mask differential expression of individual genes, we performed gene clustering after GO analysis. Seven distinct categories of gene expression profiles are shown in Figure 4. We analyzed the genes with nuclear and ribosome localization, subcategories of *Cellular Component*. The majority of nuclear distributed genes, such as Rax, Sox13, Tcf4, and Six6 (Fig. 4a), were expressed in the developmental phase and most of them reduced their expression level between the developmental and functional phases. In contrast a few of the genes in this group were preferentially expressed in the functional phase. One example of this group is Lhx3, a LIM homeodomain protein that contributes to specification of pituitary cell lineages and motor neurons [34-36]. The role of Lhx3 in the retina is unclear.

Genes in six subcategories of the *Biological Process *category were also analyzed and the results shown in figure 4. Carbohydrate metabolism genes are involved in many cellular functions and the majority of the genes from this group are highly expressed in the functional phase starting after the transition stage at PN5 (Fig. 4b). One gene in this group, B3gatl encoding beta-1, 3-Glucuronyltransferase 1, plays a role in the biosynthesis of HNK1/VC1.1/CD57 [37] which is found on both Müller glia and neuronal cells including horizontal and amacrine cells [38,39]. Different genes involved in lipid metabolism divide into two opposite expression groups by PN5 (Fig. 4c). About 9 genes have a very low level of expression in the developmental phase but significantly increase their expression levels after PN5. This group included rod specific genes Rbp3, Elovl4, and Abca4, the last two of which have been associated with macular degeneration in humans. Other genes, like Osbp2 which is found in both the macula and the peripheral neural retina of monkeys and may mediate oxysterol cytotoxicity in tissues [40], are also in this group.

Cell cycle and cell death are the critical events during retina development. The major genes involved in cell cycle processes are highly expressed in the developmental phase and their expression is significantly reduced before the transition stage (Fig. 4d). Such genes include Ccnd1, Ccnb2, Ccna2, PCNA, and Cdk4. Genes from this group are also expressed in the functional phase like Ccng2 that has been shown to be up-regulated during growth inhibition and B cell antigen receptor-mediated cell cycle arrest [41]. Expression of cell death related genes showed similar patterns as above (Fig. 4e). Genes such as Birc5, also called Survivin, which is a member of the inhibitor of apoptosis (IAP) gene family [42] and has been identified as a neuronal precursor-enriched gene [43], is expressed in the developmental phase and is suddenly lost before the transition stage at PN5.

Phosphorylation by protein kinases is a crucial process during signal transduction. Protein kinases are classified into many distinct subgroups of Tyr (Fig. 4f) and Ser/Thr (Fig. 4g) kinase families and the expression patterns of most genes in these groups are basically similar to those described above in that they are expressed on one or other side of the transition point. Only a small number of these genes are expressed in the functional phase, including Mapk14 also called p38, Clk which is a protein kinase regulated pre-mRNA splicing enzyme [44], and Nlk which is a downstream kinase of the transforming growth factor (TGF) signaling pathway and also crosstalks with STAT3 signals in mesoderm induction [45]. Interestingly, increased expression of Mapk8, also called Jnk1, that is required for apoptosis in the developing embryonic neural tube [46], occurs during the perinatal period and is reduced after the transition stage. Overall our results show that there are very few genes with high expression during the whole of retinal development and that expression of the majority of the genes becomes either reduced or increased during the transition stage. Since there are no other clear transition stages of gene expression found in this study, it suggests that the different sets of gene expression are influenced by common events during the transition stage.

### Correlation coefficient of gene expression between microarray and SAGE analysis

**Figure 5:**
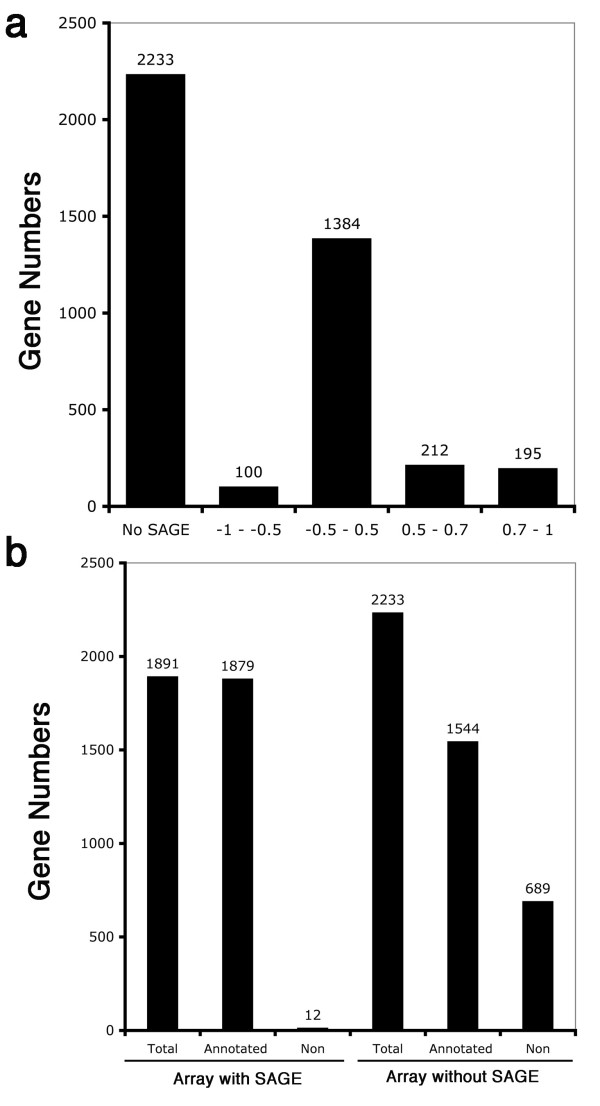
Comparison of gene expression datasets from microarray and SAGE database. a. Distribution of distinct correlation coefficient rates from the comparison of microarray and SAGE datasets. b. The annotated and non-annotated gene numbers in microarray and SAGE datasets.

The serial analysis of gene expression (SAGE) method has been used for studying gene expression during mouse retina development [47,48]. Here we compared the data from the SAGE and our microarray studies. Among more than 9,000 microarray good spots, we found 4,076 SAGE sequence tags which matched our microarray (additional file 6). In the 4,180 significantly changed genes, 2,233 genes did not have matches in the SAGE database (Fig. 5a). To have equal and comparative data from the two studies, we have reorganized the data from SAGE database and transferred the data format to approximate our microarray profiles which are presented as ratio of gene expression at each time point from the total expression levels. We first extracted the SAGE sequence tag numbers of E12, E14, E16, E18, PN0, PN2, PN4, PN6, PN10, and adult from the SAGE database [47,48] and normalized them with the total SAGE sequence tags for each gene as gene expression profiles. A correlation coefficient analysis showed that only a small population (195 genes, 10.3%) with a high expression level and retina preferential expression shows a high correlation between the SAGE and microarray studies, whereas about 5% genes (100 genes) showed a negative correlation (Fig. 5a). Since there is not perfect compatibility between the two systems and experimental designs, the main body of the gene expression profiles have a lower correlation, indicating that complementarily distinct methods will be beneficial for studies of gene expression profiles as a whole. Another cause of the lower correlation might be the poor annotation that reduces the matching rates. As shown in Figure 5b, only 0.6% of the genes (12 of 1,891) which have been annotated in the microarray were not matched with SAGE data, whereas more than 30% of the genes (689 of 2,233) which have not been annotated were not matched. A sheet combining microarray data from this study, SAGE data [47], and in situ hybridization [48] is provided as additional file 7. A total of 369 genes with in situ hybridization information can be found in our microarray database.

### Later expression genes are not restricted in rod population

**Figure 6:**
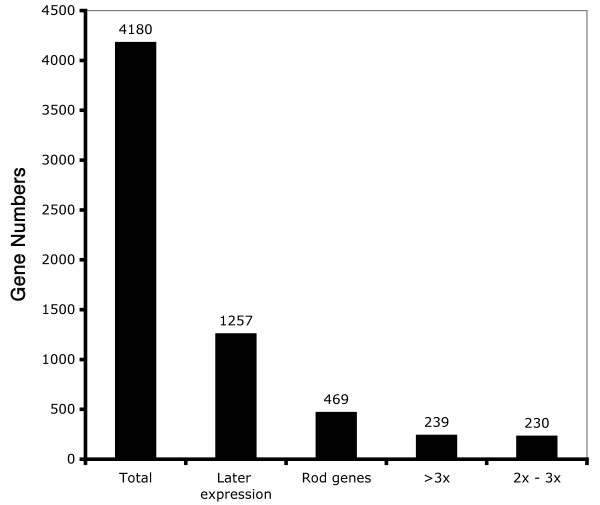
Distribution of expression gene numbers in total observed population (total), gene expression only in later phase which extracted from total (later expression), total rod expression genes which extracted from later expression genes (rod genes), preferential rod expression genes which extracted from rod genes (3x), and rod expression genes which extracted from rod genes (2x–3x).

One possible bias of the later gene expression pattern might be due to a dominant cell population, like rod photoreceptors, that are generated during postnatal retina development. To test whether this is a major contributing factor to our data we have compared the rod expressed genes, defined in our previous study by comparing gene expression in wild-type and rd1 retinas [21], with the total set of genes expressed in the later phase of this study. As shown in Figure 6, about 37% of the genes (469 of 1,257) of the later phase population were related to rods. About 20% of the genes (239 of 1,257) in this later phase were expressed with more than a 3 fold difference in expression in wild-type retina compared with rd1 retina (> 3x). These results indicate that genes expressed in the later phase are not restricted to rod photoreceptors, and that other retina cell types might also contribute to the population.

Many of the later phase expressed genes unrelated to rods may be expressed by bipolar cells and Müller glial cells that develop at the same time as rods, as suggested previously [48]. For example, Glul, glutamate-ammonia ligase in Müller glial cells and Pcp2, Purkinje cell protein 2 (L7) in bipolar cells are expressed with the same time course as genes characteristic of rod development. However, genes from the same later expression group are also typical of other cell types of retina which appear earlier in development. For example, Ext2, a gene related to heparan sulfate biosynthesis is required for axon sorting in retina ganglion cells [49] and Gnat2, a cone transducin alpha-subunit [50] show the same developmental profile as known rod genes. The developmental profile of some genes has not been described before, such as Gabral, a gamma-aminobutyric acid (GABA-A) receptor, or has been shown to be related to retinal ON-type bipolar cells [51], Müller glial cells [52], and cone photoreceptors [53]. Stxbp1, a protein implicated in vesicle trafficking and neurotransmitter release in this group is known to be highly expressed in retina [54]. These results indicate that gene expression in the later phase not only reflects late developing cell types in retina, rods, bipolar cells and Müller cells, but also includes gene expression in early developed neurons such retinal ganglion cells. An additional file 8 provides detailed information of all the later phase expression genes and their relationship with rods.

### Few genes show a peak of expression between the two developmental phases

**Figure 7:**
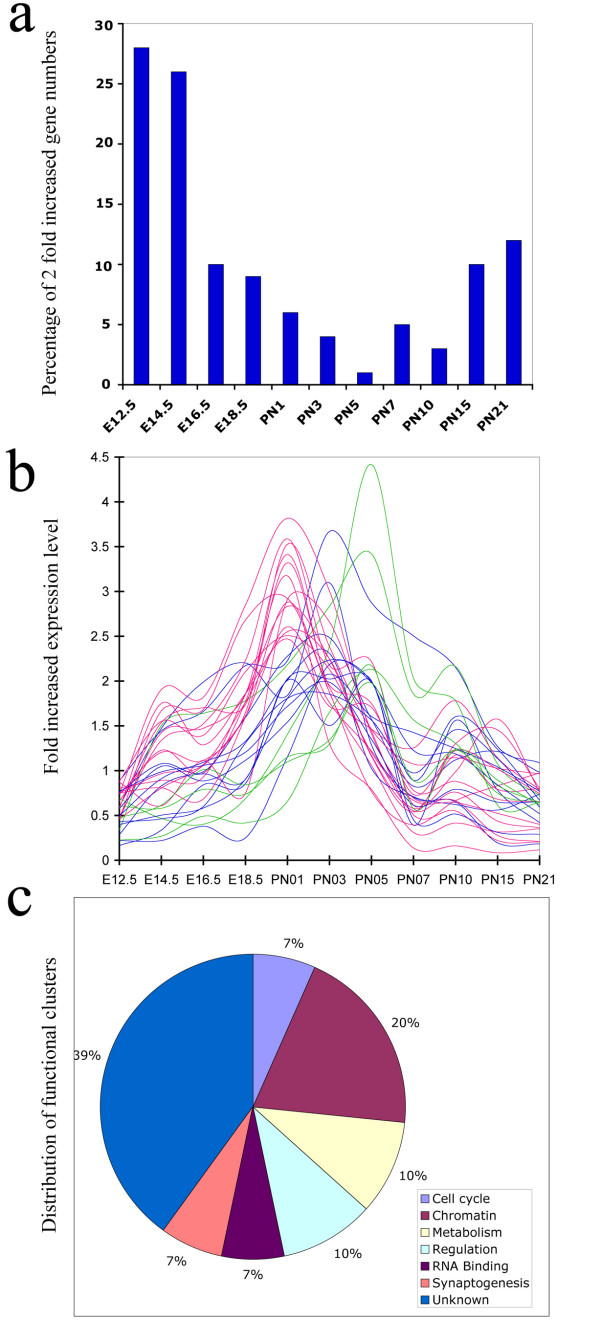
Analysis of gene expression during transition stage. a. Proportion of highly expressed genes during distinct developmental stages. b. Gene expression changes during transition stages. Red line, PN1; Blue line, PN3; and Green line, PN5. c. A pie-chart of gene distribution with functional classification

**Figure 8:**
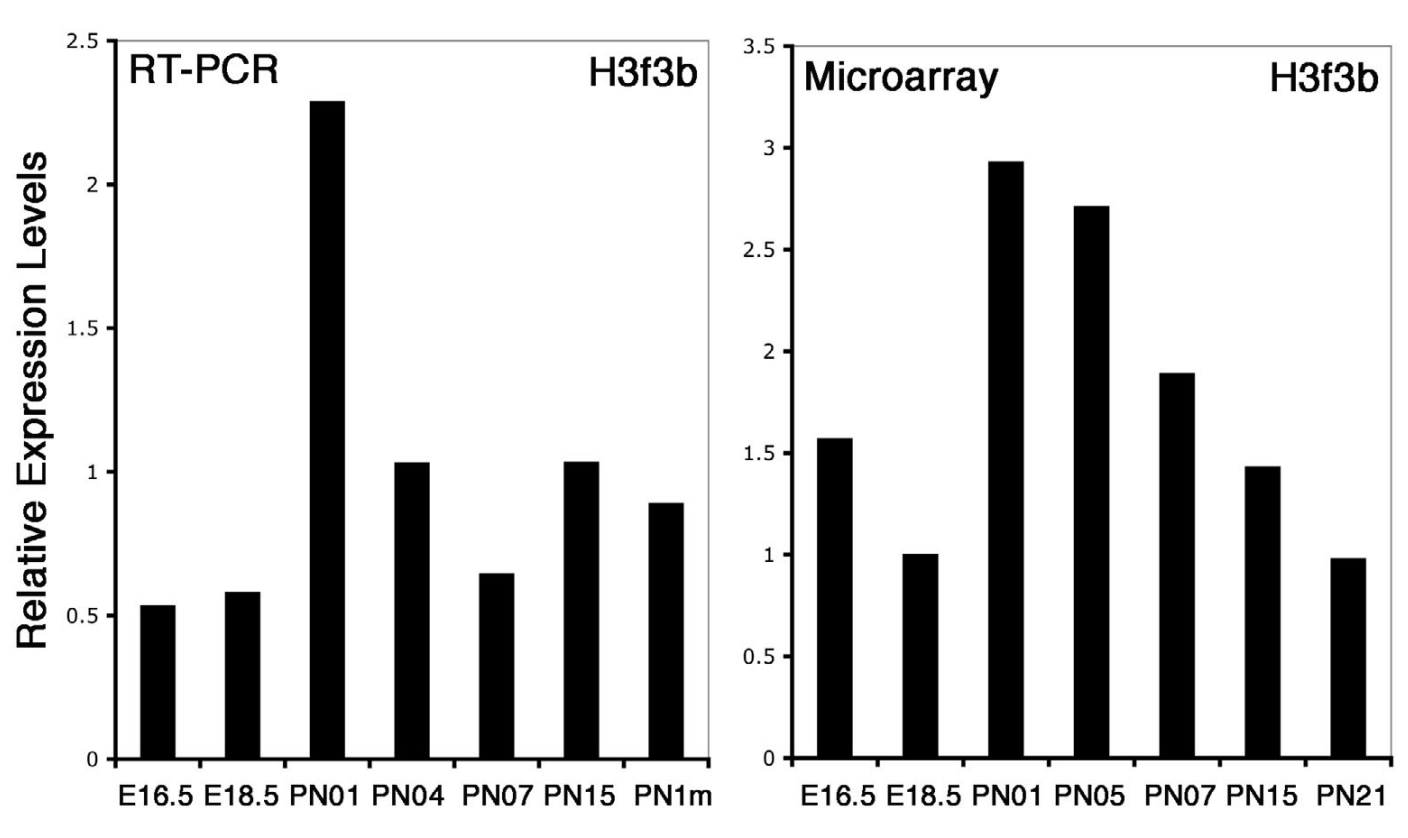
Relative expression levels of H3f3b during retina development by quantitative RT-PCR and microarray studies.

We found only a few genes whose peak of expression occurred between the two developmental phases. As shown in Figure 7a, less than 1% of the genes possessed expression levels 2 fold higher than average signals at PN5 compared with other time points. Only 31 genes were found with expression peaks in the cluster III and IV (PN1 to PN5), (Fig. 7b). As shown in additional file 9, 11 of these genes have no known function; the functions of the 20 other known genes are summarized in Figure 7c. All 20 genes belong to six functional categories, cell cycle control, chromatin structure and modeling, metabolism, gene regulation, RNA binding, and synaptogenesis. The expression levels of six of the genes were confirmed by quantitative RT-PCR using a different set of RNA from that used in the microarray experiments (Additional file 3 and Fig. 8). Although Ccnd1 and Cdk2ap1 showed a steady decrease in expression from El 6, other genes such as Fh11, Sat1, Tmpo, and H3f3b showed a close agreement between microarray and RT-PCR analysis. Among these categories, chromatin structure and modeling contained the most genes. These included H3f3b, also known as histone H3.3, which is a replacement histone and is expressed in quiescent or terminally differentiated cell [55] and Dnmt3a which encodes a de novo methyltransferase [56]. Dkk3, as a Wnt signaling antagonist is one of this group and shows highest expression in mouse brain, eye, and heart [57]. It shows differential expression during retina degeneration in rd1 mice [58] and is silenced by CpG hypermethylation in some tumor cells [59].

## Discussion

Although there have been several genome-wide studies in the eye in recent years [14-16,21,60-62], only one other has attempted a genome-wide analysis during a large segment of retina development. Using serial analysis of gene expression (SAGE), Blackshaw et al. reported gene expression during retina development but focused on a set of genes expressed by mammalian rods [47]. Dorrell et al. have shown a global gene expression analysis of the developing postnatal mouse retina [17]. Other studies have focused on much more restricted aspects of retinal development and function. For example, a microarray analysis of the transcriptional network controlled by Crx was performed by Livesey et al, suggesting that cDNA microarrays can be used successfully to define the transcriptional network in vertebrates [14]. Diaz et al used a general cDNA microarray to examine gene differential expression in the dorsal-ventral axis during mouse retina development [16]. Mu and colleagues tested gene expression in the developing mouse retina using a small-scale E14.5 retina microarray [60]. In the present study we used a microarray prepared from retinal cDNA clones which are expressed during major mouse retina developmental stages, and profiled the gene expression of retina from prenatal to postnatal stages.

We found a low correlation coefficient of gene expression during retina development between the SAGE studies [47,48] and our microarray. Possible reasons for the low correlation are; the distinct experimental design; different experimental conditions, incomplete annotation, and lower copy numbers of SAGE sequence tags which we have noted that only about 50% of the genes have more than 5 sequence copies at any one of the time points studied in comparison to our microarray data (data not shown). Since only 5% negative correlation has been found between these two methods, we suggest that complementary methods might be beneficial for the understanding of genomic changes of the retina as a whole.

From the systems biology point view, functional genomic studies of development provide a first step towards obtaining a molecular classification of developmental stages during tissue and functional unit formation. The retina serves as a useful model for a variety of forebrain derived structures. Its development has been extensively studied by many classical systems, such as histology [63], immunohistochemistry [64], and retroviral tracing [2]. Recently Blackshaw et al. showed a genomic view of retina development using SAGE and in situ hybridization methods and a taxonomic classification of developmental retina gene expression has been generated [48]. Using hierarchical clustering, we have identified 6 individual gene expression clusters during mouse retina development. Those clusters with distinct gene expression profiles, from Cluster-I (E12.5), Cluster-II (E14.5-E18.5), Cluster-III (PN1-PN3), Cluster-IV (PN5), Cluster-V (PN7-PN12) and Cluster-VI (PN15-PN21) correspond to specific stages that have been identified by other methods [2]. Among 4,180 transcripts that changed significantly during development, approximately 2/3 were expressed at high levels in the first three Clusters and then declined whereas the other 1/3 increased expression in the Cluster-V and remained at the higher levels until at least Cluster-VI. Less than 1% of the genes observed showed a peak of expression between the two phases. It appears that Cluster-IV at PN5 represents the beginning of a maturation and functional phase that correlates with the formation of the outer plexiform layer of mouse retina [32]. Further analysis of each developmental cluster is expected to have an impact on the identification of specific functional genes at distinct stages during retina development, and understanding the mechanisms by which these genes shape specific developmental events.

Through hierarchical clustering of microarray data and in silico GO gene functional analysis, we noted that distinct gene populations are expressed before and after the PN5 (Cluster-IV) even though they may be in the same GO functional clusters. Genes characteristic of a proliferative undifferentiated neuroepithelium are not expressed or are only expressed at only low levels after the transition stage, whereas most of the genes characteristic of a functional retina are highly expressed de novo from the transition stage, indicating that different sets of genes are involved at the developmental and functional stages. It is likely that use of different genes for similar functions at different times in development is a reflection of the specific networks regulating gene expression that are active at each time. This phenomenon has been shown in previous observation with individual gene studies [17,48], however the biphasic pattern have not been reported in any previous study.

Rod photoreceptors constitute the large majority of retinal cells. Most become postmitotic in the first few days after birth and undergo a large increase in gene expression as their outer segments form, beginning at about PN5. We have examined whether the biphasic expression we observed could be due simply to the onset of rod gene transcription. Although many genes selectively expressed in rods are found only in the maturation phase, this phase contains many other genes that, since they are still expressed in rd1 mutant retinas that have lost all rod photoreceptors, are probably expressed in other retinal cell types [21]. We found that less than 40% of genes in the later phase could be related to rods. While some others are known to be expressed in the other late developing cells, bipolar and Müller glial cells, yet others are characteristic of retina ganglion and cone cells, which are earlier differentiating cell types. This would suggest that most, if not all, retinal cell types have similar biphasic patterns of gene expression.

## Conclusion

This study provides a genome-wide view of common gene dynamics and a molecular classification of mouse retina development. It also provides a detailed comparison with a previous SAGE study. The results from such complementary studies will provide more accurate information for genomic studies of retina. Although many genes have distinct expression patterns with their own peaks through development, only a few genes showed peak expression between the two expression phases. Among those genes, a high frequency of cell cycle control and chromatin modification related genes were noted. We propose that large-scale changes in gene regulation during development might be related to cell cycle exit and chromatin remodeling.

## Methods

### Construction of retina tissue-specific cDNA microarray

A set of about 12,000 non-redundant sequenced mouse retina ESTs have been generated [21] from distinct development stages (E13.5, PN1, and adult) in collaboration with Dr. Soares at the University of Iowa. Purified PCR products from 9,216 cDNA clones were printed by the Yale Keck Microarray Core [65] on in-house poly-L-Lysine coated glass slides utilizing a GeneMachines Omnigrid robotic arrayer (GeneMachines).

### Mice and retina sample collection

C57BL/6j strain mice were obtained from the Jackson Laboratory (Bar Harbor, Maine). Mice and pregnant mice were euthanized at 11 designated developmental time points (E12.5, E14.5, E16.5, E18.5, PN1, PN3, PN5, PN7, PN10, PN15, and PN21). Retinas were dissected without contamination by lens, iris, cornea, or ciliary body. However, there was a very low level contamination from lens at the El2.5 stage and a small amount of retina pigment epithelium tissue could not be removed from retina at all the stages. An average of 20 to 200 retinas from each developmental stage were pooled for RNA isolation. All experiments with animals were conducted in accordance with National Institutes of Health guidelines and were approved by the Animal Care and Use Committee of Yale University School of Medicine

### RNA sample preparation

Total RNA was isolated by TRIzol (Invitrogen) and purified by a RNeasy mini kit (QIAGEN). RNA from each time point with a 280/260 ratio greater than 1.9 was used for array hybridization without amplification. Five μg total RNA from each time point was used as test samples. A mixture consisting of equal amounts of total RNA from each time point was used as control. This means that the highest expression ratio we could obtain in these experiments is no more than 11 fold higher in the test samples than in the control samples since 11 samples are pooled in the control.

### Microarray experiments and imaging

The detailed description of experimental procedures have been described previously [21]. 3DNA Submicro EX Expression Array Detection Kits (Genisphere, PA) were used for RNA labeling. A modified manufacturer's protocol was applied for all microarray experiments in this study. Slides were scanned on a GenePix 4000B scanner and the data were manipulated with GenePix Pro microarray analysis software Version 4.0 (Axon Instruments).

### Microarray data analysis

Three sets of microarray data for each time point (33 microarrays in total) were collected for microarray analysis. For developmental microarray experiments, genes were designated as changed significantly if the average changes from the three arrays were greater than 2-fold with p < 0.05 by one-way ANOVA analysis. 4,180 genes met both criteria and were analyzed in more detail. Gene Cluster and Tree View software were used for hierarchical gene and array cluster analysis [9,66]. Both gene and array clusters were performed. Gene Ontology analysis was performed for gene function clusters [33].

### SAGE data reorganization and comparison

SAGE data table [48] was download from the PLOS journal website [67]. In situ hybridization data table [48] was also download from the PLOS journal website [68]. Any SAGE entries belonging to multiple Unigene classes were removed from the table. All SAGE entries were grouped according their Unigene clusters. The numbers at same time point were summed. Finally, we generated a joint table using the same Unigene number from our microarray data, SAGE data, and in situ hybridization data. Correlation coefficient analysis is applied for the data from microarray and SAGE experiments. The equation for the correlation coefficient is:

ρx,y=Cov(X,Y)σx⋅σy
 MathType@MTEF@5@5@+=feaafiart1ev1aaatCvAUfKttLearuWrP9MDH5MBPbIqV92AaeXatLxBI9gBaebbnrfifHhDYfgasaacH8akY=wiFfYdH8Gipec8Eeeu0xXdbba9frFj0=OqFfea0dXdd9vqai=hGuQ8kuc9pgc9s8qqaq=dirpe0xb9q8qiLsFr0=vr0=vr0dc8meaabaqaciaacaGaaeqabaqabeGadaaakeaaiiGacqWFbpGCdaWgaaWcbaGaemiEaGNaeiilaWIaemyEaKhabeaakiabg2da9maalaaabaGaem4qamKaem4Ba8MaemODayNaeiikaGIaemiwaGLaeiilaWIaemywaKLaeiykaKcabaGae83Wdm3aaSbaaSqaaiabdIha4bqabaGccqGHflY1cqWFdpWCdaWgaaWcbaGaemyEaKhabeaaaaaaaa@45A0@

where:

-1 ≤ *ρ*_x*y *_≤ 1

and:

Cov(X,Y)=1n∑i=1n(xi−μx)(yi−μy)
 MathType@MTEF@5@5@+=feaafiart1ev1aaatCvAUfKttLearuWrP9MDH5MBPbIqV92AaeXatLxBI9gBaebbnrfifHhDYfgasaacH8akY=wiFfYdH8Gipec8Eeeu0xXdbba9frFj0=OqFfea0dXdd9vqai=hGuQ8kuc9pgc9s8qqaq=dirpe0xb9q8qiLsFr0=vr0=vr0dc8meaabaqaciaacaGaaeqabaqabeGadaaakeaacqWGdbWqcqWGVbWBcqWG2bGDcqGGOaakcqWGybawcqGGSaalcqWGzbqwcqGGPaqkcqGH9aqpdaWcaaqaaiabigdaXaqaaiabd6gaUbaadaaeWbqaamaabmaabaGaemiEaG3aaSbaaSqaaiabdMgaPbqabaGccqGHsisliiGacqWF8oqBdaWgaaWcbaGaemiEaGhabeaaaOGaayjkaiaawMcaamaabmaabaGaemyEaK3aaSbaaSqaaiabdMgaPbqabaGccqGHsislcqWF8oqBdaWgaaWcbaGaemyEaKhabeaaaOGaayjkaiaawMcaaaWcbaGaemyAaKMaeyypa0JaeGymaedabaGaemOBa4ganiabggHiLdaaaa@51D0@

Matched data from microarray and SAGE has been load to Microsoft Excel (2000) and the correlation coefficient was accounted between -1 to 1.

### Quantitative RT-PCR (qRT-PCR)

Different sets of RNA have been isolated from retinas at designed time points. The detail methods has been described previously [31]. The concentration of each cDNA sample was measured by Oligreen ssDNA Quantitation Kit (Invitrogen (O-11492) after digested by Ribonuclease A (R6513 Sigma) and T1 (R1003 Sigma). qRT-PCR data were normalized by the cDNA concentration. To present the qRT-PCR results in the way of our microarray experiment design, we calculated average expression level of all the observed time points for each gene from qRT-PCR results and set it as the mean of the biological expression level. The ratios of each time point's expression level to the biological mean were compared.

### Statistics

Three repeats of microarray data, each with 11 developmental points, were analyzed by one-way ANOVA test between time points to identify groups of genes with significant expression changes during these time points [69]. One-way ANOVA test showed that about 50% (4,588) of genes changes occurred within P < 0.05, about 35% (3,213) within P < 0.01, about 22% (1,994) within P < 0.001, about 13% (1,193) within P < 0.0001.

## List of abbreviations

GO: gene ontogeny

## Authors' contributions

SSZ was primarily responsible for the design, coordination, conduct, and all experiments of the studies. XYF and CJB were responsible for coordination of the studies. XX were responsible for computational data analyses and software development. SSZ and MGL were responsible for microarray experiments and analysis. MGL and HZ were responsible for statistical analysis. SSZ, MBS, and XYF were responsible for RNA collection and original initiation and generation of mouse retina ESTs. SSZ and CJB drafted the manuscript and figures. All authors read and approved the final manuscript.

## Supplementary Material

Additional File 1 A list of average intensities for each genes at all time points observed. Average relative intensities from three independent microarray experiments at each time points are listed. Average intensities from test group are highlighted with pink color and average intensities from control group are highlighted with brown color. Click here for file

Additional File 2 A comprehensive list of gene expression during retina development. The mean ratios of each gene at each time points are listed. The information are also included gene annotation, Unigene numbers, the highest and lowest level of gene expression during development and their difference ratio, F probability distribution for each gene, and the gene GO information by different categories. Click here for file

Additional File 3 Comparison of microarray and quantitative RT-PCR results. Comparison of relative expression levels of microarray and quantitative RT-PCR from ten genes are shown in this file which includes Ccnd1, Ccpg1, Cdk2ap1, Ext2, Fh11, Glu1, Rds, Rho, Sat1, and Tmpo. Click here for file

Additional File 4 Gene clusters for different developmental stages. Gene clusters with gene expression level at least 3 fold higher or lower than average expression in the different developmental stages (Cluster-I to VI). Click here for file

Additional File 5 Gene changed significantly during later postnatal developmental stages. Collection of gene changed only in the functional phase (Cluster-V and VI). Click here for file

Additional File 6 Data from microarray and SAGE studies are used for comparison. The criteria for gene collection from microarray experiments was with average changes from the three arrays were greater than 2-fold with p<0.05 by one-way ANOVA analysis. SAGE information was from , Table S2. The information provided from this file include gene annotation, Unigene numbers, gene expression in both analysis, SAGE expression information in deferent developmental stages, total SAGE tag numbers for each gene, gene expression ratio from microarray studies.

Additional File 7 A combined list of microarray, SAGE, and in situ hybridization datasets. Combined information from microarray, SAGE, and in situ hybridization database in one list. Click here for file

Additional File 8 A list of rod expression gene in the later gene expression profile. Rod expression gene data is from Zhang et al. 2005 (3x, gene expression wild-type/rd1 > 3; 2X, gene expression wild-type/rd1 > 2) to compare with the data from expressed gene in the later developmental phase. This file includes gene annotation, rod expressed genes, and gene expression levels during retina development. Click here for file

Additional File 9 Gene expression peak in Cluster-III and Cluster-IV. Genes with their highest expression peak in the Cluster-III and IV (postnatal days 1 to 5). This file includes gene accession numbers, gene name, the time expression peak, and possible functional clusters. Click here for file
