# Supplementary material for: A biphasic pattern of gene expression during mouse retina development
Source: BMC Dev Biol. 2006 Oct 17;6:48. doi: 10.1186/1471-213X-6-48 (PMC1633734; doi:10.1186/1471-213X-6-48)
Supplement: Additional File 9 — Gene expression peak in Cluster-III and Cluster-IV. Genes with their highest expression peak in the Cluster-III and IV (postnatal days 1 to 5). This file includes gene accession numbers, gene name, the time expression peak, and possible functional clusters. [file 1471-213X-6-48-S9.doc]

**Additional File 9. Gene expression peak in Cluster-III and Cluster-IV.**

|  |  |  |  |  |
| --- | --- | --- | --- | --- |
| **Accession No.** | **Unigene No.** | **Title** | **Peak** | **Function** |
| BE981938 | Mm.150686 | Bccip | PN3 | Cell cycle |
| BE951470 | Mm.273049 | Ccnd1 | PN1 | Cell cycle |
| BE985383 | Mm.332693 | Cdk2ap1 | PN3 | Cell cycle |
| BE950171 | Mm.5001 | Dnmt3a | PN3 | Chromatin |
| BE951501 | Mm.18516 | H3f3b | PN3 | Chromatin |
| BF464991 | Mm.336087 | Hmgb3 | PN1 | Chromatin |
| BE950004 | Mm.319660 | Hmgn2 | PN5 | Chromatin |
| BE952130 | Mm.2734 | Sat1 | PN5 | Chromatin |
| BE949784 | Mm.159684 | Tmpo | PN1 | Chromatin |
| BF461253 | Mm.289431 | Eef2 | PN1 | Metabolism |
| BF466792 | Mm.22505 | Hsd17b12 | PN3 | Metabolism |
| BF460832 | Mm.290692 | Tkt | PN1 | Metabolism |
| BE987967 | Mm.55143 | Dkk3 | PN1 | Regulation |
| BE987415 | Mm.142822 | Ewsh1 | PN1 | Regulation |
| BF461151 | Mm.3126 | Fhl1 | PN1 | Regulation |
| BF464663 | Mm.17898 | Cirbp | PN3 | RNA Binding |
| BE953734 | Mm.128512 | Rbm3 | PN1 | RNA Binding |
| BE949920 | Mm.268902 | Cplx2 | PN5 | Synaptogenesis |
| BE986316 | Mm.312068 | Nrxn1 | PN1 | Synaptogenesis |
| BF461452 | Mm.29476 | 2810404F18Rik | PN1 | Unknown |
| BE953578 | Mm.347721 | 2810449C10Rik | PN3 | Unknown |
| BE951365 | Mm.199223 | AA408140 | PN3 | Unknown |
| BE950035 | Mm.288602 | D230014I24 | PN3 | Unknown |
| BF464989 | Mm.358909 | D430041D05Rik | PN1 | Unknown |
| BF461098 | Mm.150838 | E130112H22Rik | PN1 | Unknown |
| BE982094 | Mm.291864 | E130113E03Rik | PN3 | Unknown |
| BF461222 | Mm.290530 | E430034L04Rik | PN5 | Unknown |
| BE996432 | None | EST | PN5 | Unknown |
| BE949642 | None | EST | PN3 | Unknown |
| BE987457 | None | EST | PN3 | Unknown |
